# Tuina for perimenopausal insomnia: A protocol for systematic review and meta-analysis

**DOI:** 10.1097/MD.0000000000028498

**Published:** 2022-01-14

**Authors:** Xiaoyu Zhi, Hongshi Zhang, Yangshengjie Liu, Ye Zhang, Jiabao Sun, Xuewei Zhao, Yuesong Yang, Peng Liu

**Affiliations:** a Department of Acupuncture and Tuina, Changchun University of Chinese Medicine, Changchun, China; b Nursing College of Changchun University of Chinese Medicine, Changchun, China; c Beihua University, Jilin, China; d Department of Acupuncture and Tuina, Affiliated Hospital of Changchun University of Chinese Medicine, Changchun, China.

**Keywords:** meta-analysis, perimenopausal insomnia, protocol, systematic review, Tuina

## Abstract

**Background:**

Insomnia in perimenopausal women has a negative impact on quality of life and increases health care costs. With the increasing incidence of the disease, Tuina has been accepted by perimenopausal women. The purpose of this study is to explore the effectiveness and safety of Tuina for perimenopausal insomnia.

**Methods:**

We will search 9 electronic databases: Chinese Biomedical Literature Database, Cochrane Library, China National Knowledge Infrastructure, EMBASE, MEDLINE, Web of Science, PubMed, Wan fang, Chinese Scientific Journal Database, and 1 clinical trials register platform: WHO International Clinical Trials Registry Platform. All relevant randomized controlled trial using Tuina for perimenopausal insomnia will be included. Two reviewers will independently screen date, and meta-analysis will be performed with RevMan (V5.3.5) software.

**Results:**

This study will provide an evidence of Tuina for perimenopausal insomnia.

**Conclusion:**

This study will provide a reliable evidence for the evaluation of the efficacy and side effects of Tuina in the treatment of perimenopausal insomnia.

**PROSPERO registration number:**

CRD42021259017.

**Ethics and dissemination:**

This systematics review will evaluate the efficacy and safety of tuina in the treatment of perimenopausal insomnia. Since all the data included were published, the systematic review did not require ethical approval.

## 1 Introduction

Perimenopause is associated with increased frequency of sleep disturbances, and insomnia is one of the most frequently reported symptoms among perimenopausal women.^[1,2]^ There are many factors can contribute to insomnia during menopause, including hot flashes, anxiety and depression, behavioral, and psychosocial factors.^[3]^ Insomnia in perimenopausal women is more severe and lasts longer than in non-menopausal women.^[4,5]^ Taking care of menopausal women's health is crucial, as they end up exhibiting relatively poor physical and mental health and a reduced quality of life if they suffer from chronic sleep disorders.^[6,7]^ Current treatment options for perimenopausal insomnia include nonpharmacological and pharmacological treatments, including cognitive–behavioral therapy for insomnia, complementary and alternative medications, hormone replacement therapy, sedative hypnotics, antidepressants, and continuous positive airway pressure.^[5,8–10]^ Tuina as an nonpharmacological and non-invasive therapy^[11]^ accepted by more and more patients. The purpose of this study is to evaluate the effectivity and safety of Tuina on perimenopausal insomnia.

## 2 Methods and analysis

Our protocol should be reported basing on the preferred reporting items for systematic reviews and meta-analyses protocols (PRISMA-P) statement guidelines. And has been registered in the PROSPERO (CRD42021259017).

### 2.1 Inclusion criteria

#### 2.1.1 Types of studies

All relevant randomized controlled trial using Tuina for perimenopausal insomnia will be included.

#### 2.1.2 Types of patients

Women who meet insomnia in perimenopause, and are clearly diagnosed as peimenopausal insomnia will be included.

#### 2.1.3 Types of intervention

All randomized controlled trials with Tuina therapy will be included.

#### 2.1.4 Outcomes

Main outcome is sleep quality values. Additional outcomes include hormone levels, side effects caused by tuina, and traditional Chinese medicine symptom changes.

### 2.2 Database search strategy

We will search Chinese Biomedical Literature Database, Cochrane Library, China National Knowledge Infrastructure, EMBASE, MEDLINE, Web of Science, PubMed, Wan fang, Chinese Scientific Journal Database, and 1 clinical trials register platform: WHO International Clinical Trials Registry Platform. The search terms including perimenopausal insomnia, perimenopausal sleep disorder, perimenopausal sleep disturbance, Tuina, massage, massage therapy, etc. The search strategies for PubMed are listed in Table 1.

**Table 1:** Search strategy for PubMed.

No	Search terms
#1	massage. ti, mesh.
#2	Tuina. ti, ab.
#3	massage therapy. ti, ab.
#4	massage therapies. ti, ab.
#5	Zone Therapy. ti, ab.
#6	Zone Therapies. ti, ab.
#7	or #1-#6
#8	perimenopausal sleep disturbance.ti, ab.
#9	perimenopausal insomnia. ti, ab.
#10	perimenopausal sleep disorder. ti, ab
#11	or #8-#10
#12	randomized controlled trial. pt.
#13	controlled clinical trial. pt.
#14	randomized. ab.
#15	randomly. ab.
#16	trial. ab.
#17	or #12-#16
#18	exp animals/not humans. sh.
#19	#7 and #11 and #17 and #18

### 2.3 Data collection and analysis

#### 2.3.1 Study selection and date extraction

**Figure 1:**
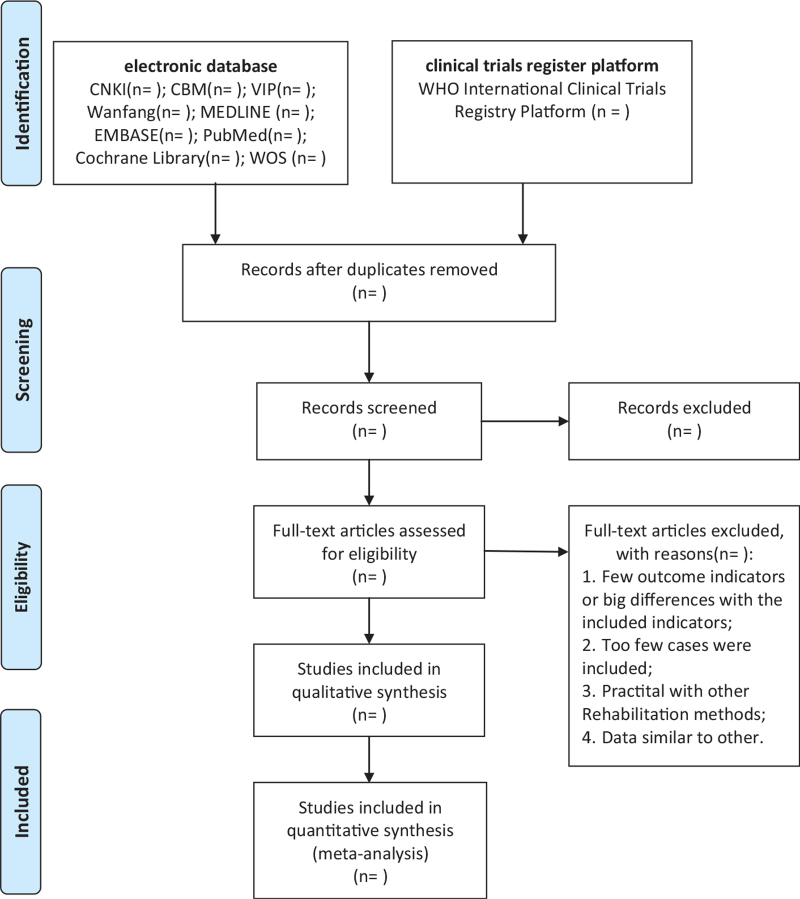
PRISMA flow diagram of study and exclusion. PRISMA = preferred reporting items for systematic reviews and meta analyses.

Two researchers (JBS and XWZ) will independently perform as selection, data extraction, and quality assessment. First, they will eliminate duplicate articles with EndNote software (V. X9.0; https://endnote.com/product-details), they will screen literature with inclusion and exclusion criteria independently. Afterward, through reading titles and abstracts, literature that is obviously not applicable will be deleted. Finally, included articles will be chosen by screening the full articles. The screening process is listed in Fig. 1. When different opinions generate between the 2 reviewers and cannot agree on through consultations, the third reviewer (YSY) will make the final decision. The data such as study design, participant characteristics, interventions both tuina, and the control intervention, results will be extracted and recorded in an electronic text. The extraction will be completed independently by 2 reviewers (YZ and YSJL) and the information will be rechecked crossly. Divergence will be made by the third author (PL) though discussion.

#### 2.3.2 Assessment of risk of bias and reporting of study quality

Two independent reviewers (HSZ and XYZ) will assess the risk of bias adopt the Cochrane risk of bias tool and complete the STRICTA checklist. And, the Jadad scale will be used to estimate the study quality.

#### 2.3.3 Measures of treatment effect

Mean differences (MDs) with 95% confidence intervals (95% CIs) will present as the continuous data. Also risk ratio (RR) will be the expression of dichotomous data.

#### 2.3.4 Unit of analysis issues

According to the outcomes, sleep quality values will be pooled to together, and the secondary outcomes including biochemical indicators, total scores on the insomnia severity index, and traditional Chinese medicine symptom changes also be analyzed, respectively.

#### 2.3.5 Management of missing data

If the information is missing or incomplete, we will attempt to contact the original author. If it cannot be supplemented, malformed data will be removed.

#### 2.3.6 Assessment of heterogeneity

Heterogeneity assessment depends on *I*^2^. *I*^2^ < 25% means negligible heterogeneity, 25% ≤ *I*^2^ < 50% means mild heterogeneity, 50% ≤ *I*^2^ < 75% means moderate heterogeneity, and *I*^2^ ≥ 75% means high heterogeneity.

#### 2.3.7 Assessment of reporting biases

Funnel plots will be used to assess reported bias when >10 trials are included. Its symmetry explains these deviations. If the funnel plot is symmetrical, no bias is reported, while asymmetry implies the presence of bias.

#### 2.3.8 Data synthesis

Quantitative analysis will be implemented using RevMan software (Version 5.3; https://community.cochrane.org/help/tools-andsoftware/revman-5) with 95% CI. The average change for each major and minor result will be combined. In addition, if the data are not suitable for quantitative analysis, qualitative description will be used.

#### 2.3.9 Subgroup analysis

Subgroup analysis will be based on difference of massage forms, participant conditions, and controls.

#### 2.3.10 Sensitivity analysis

Sensitivity analysis will be performed based on heterogeneity and predefined criteria.

## 3 Discussion

Perimenopausal insomnia is associated with subjective factors such as anxiety and depression, as well as objective factors such as income and education environment.^[12–13]^ Insomnia is the core symptom of perimenopause, the prevalence of sleep disorders increases significantly during and after menopause.^[14–18]^ Therefore, we will systematically review the efficacy of Tuina in the treatment of perimenopausal insomnia and provide new ideas for clinical.

## Author contributions

Xiaoyu Zhi and Peng Liu had the original idea of this work and drafted the protocol. The search strategy was developed by all the authors and will be performed by Xiaoyu Zhi, Hongshi Zhang, Yangshengjie Liu, Ye Zhang, Jiabao Sun, etc. Peng Liu proposed some advice for design and revision. Jiabao Sun, Xuewei Zhao, and Yuesong Yang independently collected the eligible studies. Ye Zhang, Yangshengjie Liu, and Peng Liu completed the extraction independently. Hongshi Zhang and Xiaoyu Zhi assessed the bias risk and dealt with missing data. All the authors participated in this study critically revised the final version of the manuscript and confirmed the publication of this protocol.

**Conceptualization:** Xiaoyu Zhi, Peng Liu.

**Data curation:** Yangshengjie Liu, Ye Zhang, Peng Liu.

**Formal analysis:** Xiaoyu Zhi, Hongshi Zhang.

**Funding acquisition:** Hongshi Zhang, Peng Liu.

**Investigation:** Jiabao Sun, Xuewei Zhao, Yuesong Yang.

**Methodology:** Xiaoyu Zhi, Hongshi Zhang.

**Supervision:** Peng Liu.

**Validation:** Xiaoyu Zhi, Hongshi Zhang, Peng Liu.

**Writing – original draft:** Xiaoyu Zhi.

**Writing – review & editing:** Xiaoyu Zhi, Hongshi Zhang, Peng Liu.
